# The tumour-promoting receptor tyrosine kinase, EphB4, regulates expression of Integrin-β8 in prostate cancer cells

**DOI:** 10.1186/s12885-015-1164-6

**Published:** 2015-03-22

**Authors:** Inga Mertens-Walker, Bruno C Fernandini, Mohanan SN Maharaj, Anja Rockstroh, Colleen C Nelson, Adrian C Herington, Sally-Anne Stephenson

**Affiliations:** 1 Institute of Health and Biomedical Innovation, Queensland University of Technology, Translational Research Institute, 37 Kent Street, Woolloongabba, Queensland 4102 Australia; 2 Australian Prostate Cancer Research Centre – Queensland, Princess Alexandra Hospital, Woolloongabba, Queensland 4102 Australia

**Keywords:** EphB4, Prostate cancer, Integrin-β8

## Abstract

**Background:**

The EphB4 receptor tyrosine kinase is overexpressed in many cancers including prostate cancer. The molecular mechanisms by which this ephrin receptor influences cancer progression are complex as there are tumor-promoting ligand-independent mechanisms in place as well as ligand-dependent tumor suppressive pathways.

**Methods:**

We employed transient knockdown of *EPHB4* in prostate cancer cells, coupled with gene microarray analysis, to identify genes that were regulated by *EPHB4* and may represent linked tumor-promoting factors. We validated target genes using qRT-PCR and employed functional assays to determine their role in prostate cancer migration and invasion.

**Results:**

We discovered that over 500 genes were deregulated upon *EPHB4* siRNA knockdown, with integrin β8 (*ITGB8*) being the top hit (29-fold down-regulated compared to negative non-silencing siRNA). Gene ontology analysis found that the process of cell adhesion was highly deregulated and two other integrin genes, *ITGA3* and *ITGA10*, were also differentially expressed. In parallel, we also discovered that over-expression of *EPHB4* led to a concomitant increase in *ITGB8* expression. *In silico* analysis of a prostate cancer progression microarray publically available in the Oncomine database showed that both *EPHB4* and *ITGB8* are highly expressed in prostatic intraepithelial neoplasia, the precursor to prostate cancer. Knockdown of *ITGB8* in PC-3 and 22Rv1 prostate cancer cells *in vitro* resulted in significant reduction of cell migration and invasion.

**Conclusions:**

These results reveal that EphB4 regulates integrin β8 expression and that integrin β8 plays a hitherto unrecognized role in the motility of prostate cancer cells and thus targeting integrin β8 may be a new treatment strategy for prostate cancer.

**Electronic supplementary material:**

The online version of this article (doi:10.1186/s12885-015-1164-6) contains supplementary material, which is available to authorized users.

## Background

Erythropoietin-producing hepatocellular (Eph) Type-B receptor 4 (EphB4) is part of the largest family of membrane-bound receptor tyrosine kinases (RTK) which consists of 14 different receptors which are classed as EphA or EphB. Their ligands, the ephrins, are also cell membrane-bound, either *via* glycosylphosphatidylinositol (GPI)-linkage (ephrin-A ligands) or transmembrane-embedded (ephrin-B ligands). Interaction between Eph receptors and their ligands normally takes place *in trans* through the binding of 2 ligands on one cell to 2 receptors on an adjacent cell forming a heterotetramer that is the basic complex required for signaling. EphB4 plays an important role in cell signaling and is also involved in regulating cell morphology, adhesion, migration and invasion through modification of the cell’s actin cytoskeleton and by influencing the actions of integrins [1]. Moreover, depending on the cell-environment conditions, EphB4 demonstrates the ability to be both a tumor promoter, when over-expressed and in the absence of stimulation by its sole cognate ligand, ephrin-B2, as well as a tumor suppressor stimulated by ephrin-B2 [2-6]. EphB4 is overexpressed in 66% of prostate cancer clinical samples and has been implicated in prostate cancer development and progression [2,7]. It has been shown using targeted siRNA sequences that knockdown of EphB4 in prostate cancer causes a significant reduction in cell motility *in vitro* and tumor growth *in vivo* [5]. However, the mechanisms by which removal of EphB4 exerts these effects are largely unknown. To date, no study has investigated the broader consequences on gene expression of siRNA-mediated knockdown of *EPHB4* in prostate cancer. Therefore, we sought to determine the genome-wide changes upon transient knockdown of *EPHB4* in a ligand-independent context in the prostate cancer cell line LNCaP.

Through gene expression analysis following *EPHB4* knockdown, validation of the microarray data, and EphB4 over-expression, we have determined that EphB4 regulates the expression of integrin β8 in prostate cancer cell lines.

## Methods

### Cell culture

All cell lines were purchased from the American Type Culture Collection (Manassas, VA). LNCaP, PC3 and 22Rv1 prostate cancer cells were cultured in RPMI 1640 (Life Technologies, Mulgrave, VIC, Australia) supplemented with 10% fetal calf serum (FCS). EphB4 over-expressing stable 22Rv1 cell lines, together with vector-only (VO) and parental 22Rv1 cells, were generated as described previously [2].

### siRNA transfection

Lipofectamine 2000 (Life Technologies) was used to transiently transfect LNCaP cells with 10 nM of *EPHB4* siRNAs (SI00288589, SI04435053; Qiagen, Chadstone, VIC, Australia) or PC3 and 22Rv1 cells with 100 nM of *ITGB8* siRNAs (SI00034454, SI03066623, Qiagen). The AllStars non-silencing negative control siRNA (Qiagen) was used at the same concentration as gene-specific siRNAs for all experiments. After 48 h, RNA from both the siRNA-treated cells and the EphB4 over-expressing cells was extracted using Trizol (Life Technologies).

### Microarray gene expression profiling

Triplicate samples of *EPHB4* siRNA knockdown and respective control siRNA transfected LNCaP cells were extracted for RNA and prepared for microarray profiling, which was performed on a custom Agilent 4 × 180 K oligo array (VPCv3 ID:032034, GEO GPL16604, Agilent Technologies, Mulgrave, VIC, Australia). This microarray contains the Agilent 44 K (ID:014850) probe set incorporating human gene expression protein-coding probes as well as non-coding probes; with the probes targeting exonic regions, 3′UTRs, 5′UTRs, as well as intronic and intergenic regions [8]. RNA was isolated with Trizol (Life Technologies), further purified using an RNeasy Mini Kit (Qiagen) with DNAse treatment according to the manufacturer’s protocol. RNA samples were analyzed by a Bioanalyzer (Agilent) to ensure the RNA was of high quality. RNA (100 ng) from each group was amplified and labelled using the Low Input Quick Amp Labeling Kit (Agilent) and the protocol for One-Color Microarray-Based Gene Expression Analysis. The input RNA was reverse transcribed into cDNA, using an oligo-dT/T7-promoter primer which introduces a T7 promoter region. The subsequent *in vitro* transcription uses a T7 RNA polymerase, which simultaneously amplifies target material into cRNA and incorporates cyanine 3-labeled CTP. cDNA synthesis and *in vitro* transcription were both performed at 40°C for 2 h. The labelled cRNA was then purified with Qiagen’s RNeasy mini-spin columns and quantified using a Nanodrop-1000 (Thermo Scientific, Waltham, MA, USA). cRNA (1650 ng) from each sample was loaded onto the 4x180 K custom microarray and allowed to hybridize at 65°C for 17 h. The arrays were scanned using an Agilent Microarray Scanner G2565CA.

### Microarray data analysis

The microarray data were processed with Agilent Feature Extraction Software (v10.7). A quantile between-array normalization was applied and differential expression was determined using an unpaired T-test, asymptotic p-value and a 2-fold cut-off (GeneSpring GX11, Agilent Technologies). The gene expression levels are presented as fold change. Genes that were significantly different between two groups were identified with a p value of < =0.05, and an average fold change of > = 2.

Normalized gene expression data of the experiment are Minimum Information About a Microarray Experiment (MIAME) and have been submitted to Gene Expression Omnibus (GEO) with the accession number GSE61800.

### Quantitative real-time PCR

Extracted RNA was reverse transcribed using Superscript III reverse transcriptase according to the manufacturer’s instructions (Life Technologies). Quantitative RT-PCR for *EPHB4* expression was performed in triplicate using a TaqMan Gene Expression Assay (*EPHB4*: Hs00174752_m1 Life Technologies) and TaqMan Universal PCR Master Mix, No AmpErase UNG (Life Technologies) on a Rotor-Gene 6000 (Qiagen). The endogenous reference gene hydroxymethylbilane synthase (*HMBS*: Hs00609297_m1) was used for normalization and results are expressed as the *EPHB4*:*HMBS* ratio. For integrin gene expression analysis the SYBR Master Mix (Life Technologies) was used with *GAPDH* as a housekeeping gene. Primer sequences are listed in Table 1.

**Table 1:** **Primer sequences for semi**-**quantitative RT**-**PCR and qRT**-**PCR**

Gene	Forward primer 5’ to 3’	Reverse primer 5’ to 3’	Product size (bp)
***ITGA3***	AGCGCTACCTGCTCCTGGCT	GGGCAGTGAGTGGGCACAGG	99
***ITGA10***	CTGACAGGTCTCTGCTCCCCC	CCATCGCTGTCCACCCCCAA	120
***ITGB8***	TGTGTGCTGGGCATGGAGAGTGT	CAGTGCTGGGCTGCTGCTGAA	100
***ITGAV***	CATCTGTGAGGTCGAAACAGG	TGGAGCATACTCAACAGTCTTTG	137
***EPHB4***	TCAATGTCACCACTGACCGAGAGGTA	GGTATTTGACCTCGTAGTCCAGCACA	141
***GAPDH***	CTGCACCACCAACTGCTTAG	GTCTTCTGGGTGGCAGTGAT	108

### Semi-quantitative RT-PCR

RT-PCR analysis was carried out using standard conditions with primers as shown in Table 1). PCR conditions were as follows: initial denaturation 94°C for 15 min then 94°C for 30s; 60°C for 30s; 72°C for 30s repeated 35 times, final elongation 72°C for 10 min. PCR products were analyzed on a 2% Tris-acetate agarose gel containing 0.01% ethidium bromide and photographed using a Gel doc system (Syngene, MD, USA).

### SDS-PAGE and western blotting

Cells were lysed with ice-cold RIPA buffer (50 mM Tris pH 7.4, 1% Triton X-100, 0.5% sodium deoxycholate, 150 mM NaCl, 2 mM EDTA, 1 mM sodium orthovanadate, 1 mM NaF, 1 mM PMSF, 10 μg/ml aprotinin) supplemented with protease inhibitors (Complete Mini-EDTA-free tablets; Roche, Castle Hill, NSW, Australia). Protein lysates were mixed at 4°C for 15 min, then insoluble proteins removed by centrifugation at 14,000 rpm. Protein concentrations were determined using the BCA Protein Assay Kit (Pierce, Rockford, IL, USA). Protein samples (20-50 μg) were separated on SDS-PAGE gels (4% stacking and 10% separating) before proteins were transferred to a nitrocellulose membrane using a wet transfer system (Bio-Rad, Hercules, CA, USA) in transfer buffer (20% methanol, 0.1 mM Tris, 80 mM glycine) at 100 V for 90 min. After blocking with 5% skim milk in TBST (Tris buffered saline with Tween-20:- 8 g/L NaCl, 3 g/L Tris, 0.2 g/L KCl, 0.2% Tween-20, pH 7.4) for 1 h, blots were probed with the primary antibody in 5% BSA/TBST (anti-EphB4 H-200, Santa Cruz Biotechnology, CA, USA) [9] or 5% skim milk/TBST (anti-ITGB8 ab80673, Abcam, Melbourne, VIC, Australia) overnight at 4°C before incubation with horseradish peroxidase-labelled secondary antibody anti-rabbit IgG (Sigma-Aldrich, Castle Hill, NSW, Australia) in 5% skim milk/TBST for 1 h at room temperature. Chemiluminescence was detected with Amersham™ ECL Plus Chemiluminescence kit (GE Healthcare, Silverwater, NSW, Australia) following the manufacturer’s recommendation, with a 10-30 sec exposure to SuperRX X-ray film (Fuji Film Corporation, Japan).

### Migration assay

PC3 cells were seeded into 96-well Essen Bioscience ImageLock plates (Essen BioSciences, Ann Arbor, MI, USA) at 2 x 10^4^ cells per well in four replicates and allowed to proliferate for 24 h until they had formed a complete monolayer. Cells were then transfected with ITGB8 siRNA and allowed to grow for a further 24 h. The 96-well WoundMaker (Essen Bioscience) was then used to create a cell-free zone in the monolayer before the plates were placed in the IncuCyte ZOOM (Essen Bioscience). Migration into the cell free zone was determined after 24 h and quantified as relative wound density.

### Invasion assay

Pre-coated growth-factor reduced Matrigel cell culture inserts (24-well, pore size 8 μm; BD Biosciences, San Jose, CA, USA) were seeded with serum-deprived 5 x 10^5^ PC3 or 22Rv1 vector-only cells (22Rv1-VO) or 22Rv1 EphB4 over-expressing cells (22Rv1-B4) and transfected with 100 nM *ITGB8* siRNA or non-silencing AllStars siRNA (Qiagen) in 0.1% FCS-containing medium. Medium containing 10% FCS was used as chemo-attractant. Cells were incubated for 22 h and cells that had not invaded were removed from the upper chamber using a cotton swab. Membranes were excised and mounted on glass slides with ProLong Gold Antifade containing 4, 6-diamidino-2-phenylindole, dihydrochloride (DAPI) (Life Technologies) for visualization of the nuclei of cells that had invaded through the Matrigel to the underside of the membrane. Nuclei were counted in five random fields at 20 X magnification using an Olympus epifluorescent microscope.

### Adhesion assay

22Rv1-VO or –B4 cells, or their negative siRNA-control or *ITGB8* siRNA counterparts (1 x 10^5^ cells), were seeded into triplicate wells in a Vitronectin pre-coated 96 well adhesion assay plate, and an adhesion assay was carried out according to the manufacturer’s instructions (Merck Millipore, VIC, Australia).

### Statistics

Statistical analysis was carried out using IBM SPSS software package by employing the ANOVA analysis followed by Fisher’s least significant difference post hoc test or Student’s t-test with p < 0.05 considered significant.

## Results

### *EPHB4* down-regulation results in differential gene expression in LNCaP cells

In an effort to characterize the contribution of EphB4 to regulating gene expression in prostate cancer, endogenously EphB4-expressing LNCaP cells were transfected with *EPHB4* specific siRNAs and compared to negative non-silencing siRNA cells using gene expression profiling. Quantitation of Western immunoblots using Image J confirmed knockdown of *EPHB4* averaged 80.25 ± 8.96%. Using a 2-fold cut off, 260 genes were up-regulated and 300 genes were found to be significantly down-regulated when *EPHB4* siRNA transfectants were compared to the negative controls (Additional file 1: Figure S1). Among the top ten down-regulated genes were *ITGB8* (29.4 fold), *EIF4E3* (10.3 fold) and *SASH1* (6.8 fold) (Table 2). On the other hand, the top ten up-regulated genes included *MYO VI* (5.7 fold), *MMP12* (4.8 fold) and *CYP11A1* (4.2 fold) (Table 3). Gene ontology screening highlighted the biological process of cell adhesion as being significantly de-regulated upon *EPHB4* knockdown (Table 4). Amongst the genes involved in cell adhesion were three integrin molecules that were de-regulated (Table 4) including the top down-regulated gene, *ITGB8.* We therefore chose to further investigate the relationship between EphB4 and those three integrins - *ITGB8*, *ITGA3* and *ITGA10*.

**Table 2:** **Top ten significantly down-regulated genes in LNCaP cells after** ***EPHB4*** **siRNA knockdown**

Fold change	Gene symbol	Description	Function
-**29.4**	*ITGB8*	Integrin beta 8	Cell-cell and cell-extracellular matrix interactions
**-10.5**	*LOC100287846*	Patched 1 pseudogene	Uncharacterized
**-10.5**	*PROM1*	Prominin 1/CD133	Transmembrane glycoprotein, stem cell marker
**-10.3**	*EIF4E3*	Eukaryotic translation initiation factor 4E family member 3	Recruits mRNA to ribsosome
**-8.8**	*GBP3*	Guanylate Binding Protein 3	Binding of guanidine nucleotides
**-8.4**	*DOCK10*	Dedicator of Cytokinesis 10	Guanine nucleotide exchange factor
**-7.9**	*C14orf38*	Open reading frame	Uncharacterized
**-6.9**	*TMC7*	Transmembrane Channel-like 7	Multi-pass membrane protein
**-6.8**	*SASH1*	SAM and SH3 domain Containing 1	Candidate tumour suppressor in breast cancer
**-5.8**	*FSD2*	Fibronectin type III and SPRY domain containing 2	Uncharacterized

**Table 3:** **Top ten significantly up-regulated genes in LNCaP cells after** ***EPHB4*** **siRNA knockdown**

Fold change	Gene symbol	Description	Function
**5.7**	*MYO6*	Myosin VI	Actin-based motor protein
**4.8**	*MMP12*	Matrix-metallopeptidase 12	Cleavage of extra-cellular matrix
**4.4**	*LOC100131726*	HCC-related HCC-C11_v3	Miscellaneous RNA
**4.3**	*TDRD7*	Tudor Domain containing 7	Component of cytoplasmic RNA granules
**4.3**	*OSMR*	Oncostatin M receptor	Type I cytokine receptor
**4.3**	*APOBEC3H*	Apolipoprotein B mRNA editing enzyme, catalytic polypeptide-like 3H	Antiretroviral mRNA editing enzyme
**4.2**	*CYP11A1*	Cytochrome P450, family 11, subfamily A, polypeptide 1	Conversion of cholesterol to pregnolone
**4.2**	*LRRTM3*	Leucine Rich Repeat Transmembrane Neuronal 3	Implicated in neuronal disorders
**4.1**	*GCOM1*	GRINL1A complex locus 1	Read-through transcription between the MYZAP (myocardial zonula adherens protein) and POLR2M (polymerase (RNA) II (DNA directed) polypeptide M
**3.9**	*TBC1D8B*	TBC1 domain family, member 8B	Uncharacterized

**Table 4:** **Significantly enriched gene ontology process in LNCaP cells after** ***EPHB4*** **siRNA knockdown**

GO biological process	Genes involved
Cell adhesion:	*ACHE; CD34; CDH19; CLDN10; CNTN6; COL5A1; DLC1; DSC1; DSCAM; FEZ1; FRAS1; ITGA10; ITGA3; ITGB8; JAM2; LAMA4; MTSS1; NELL2; NLGN3; NRCAM; PKD2; STAB2; TGFBI; TNC; TNF; VCL*
GO:0007155	

### Integrins are significantly de-regulated in response to changing EphB4 levels in prostate cancer cells

**Figure 1:**
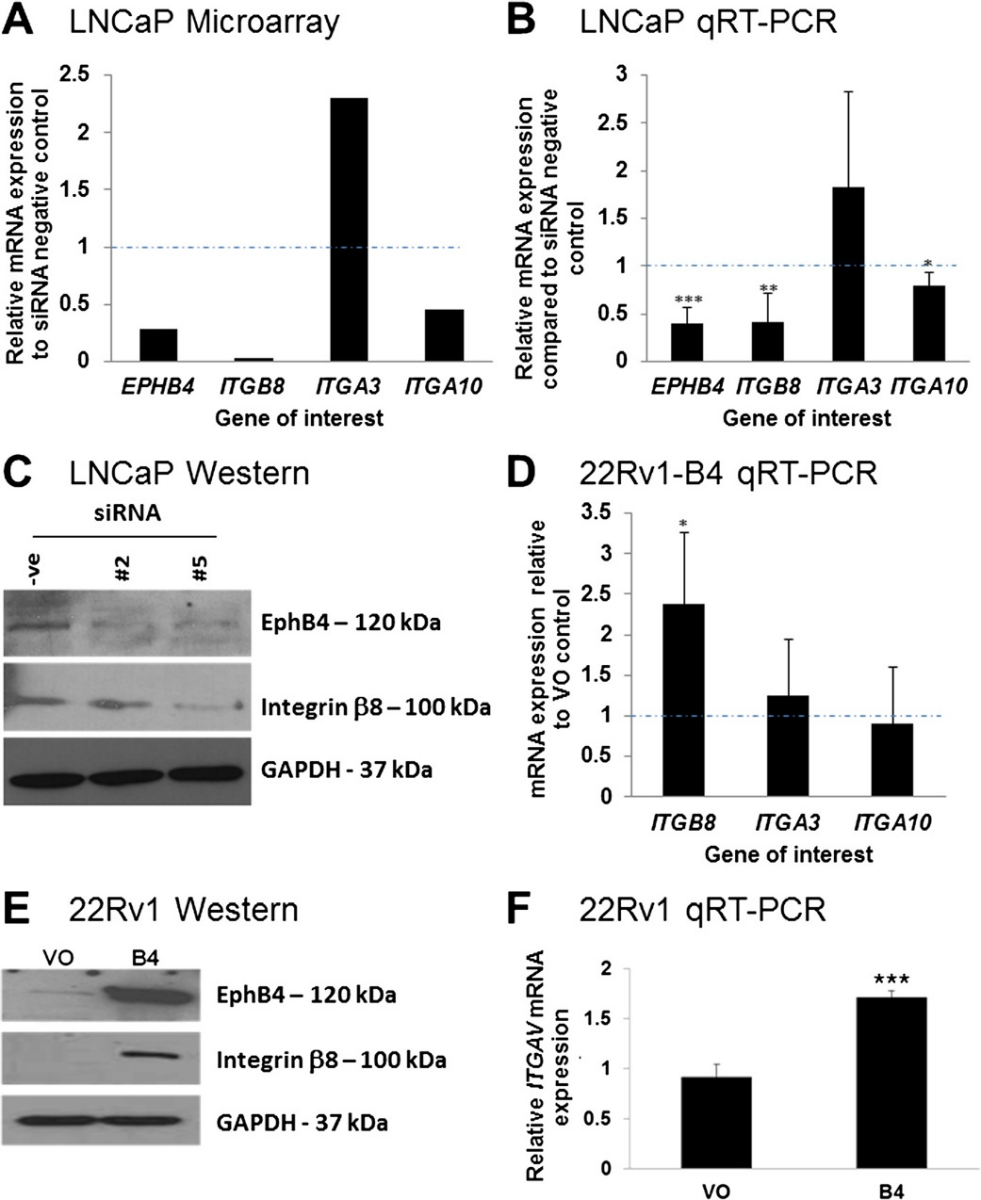
**Integrins are significantly de**-**regulated in response to changing EphB4 levels in prostate cancer cells. A)** Relative gene expression of *EPHB4*, *ITGA3*, *ITGA10* and *ITGB8* after siRNA knockdown of *EPHB4* in LNCaP cells as identified on the cDNA microarray, compared to control negative siRNA. Dotted line indicates normalized level of negative siRNA control. **B)** Relative gene expression normalized to *GAPDH* of *EPHB4*, *ITGA3*, *ITGA10* and *ITGB8* after siRNA knockdown of *EPHB4* in LNCaP cells as determined by qRT-PCR (independent experiments from results shown in **A**). Dotted line indicates normalized level of negative siRNA control. **C)** Western blotting analysis showing integrin β8 and EphB4 protein levels in LNCaP cells that have been transfected with two different siRNAs (#2 and #5) targeting *EPHB4*. GAPDH was used to normalize for loading. **D)** Relative gene expression of *ITGA3*, *ITGA10* and *ITGB8*, normalized to *GAPDH*, in stably over-expressing 22Rv1-B4 cells as determined by qRT-PCR. Dotted line indicates normalized level of negative siRNA control. **E)** Western blotting analysis showing integrin β8 and EphB4 protein levels in 22Rv1-B4 over-expressing prostate cancer cells. GAPDH was used as a loading control. **F)** Relative gene expression of *ITGAV* in stably over-expressing 22Rv1-B4 compared to VO (vector only) cells as determined by qRT-PCR. QRT-PCR experiments were carried out in triplicate and with three biological replicates. Western blotting experiments were carried out three times and representative cropped blots are shown. Graphs are presented with ± SD. *** *p* < 0.005, ** *p* < 0.01, * *p* < 0.05.

The microarray analysis revealed significant quantitative changes in *ITGB8* and *ITGA10* (down-regulated) and *ITGA3* (up-regulated) (Figure 1A). To confirm these data we employed real-time PCR analysis on a different set of LNCaP transfectants. Upon *EPHB4* knockdown, *ITGB8* and *ITGA10* were significantly down-regulated (Figure 1B). *ITGA3* however, was unchanged (Figure 1B). Knockdown of *EPHB4* with two different siRNAs also resulted in a reduction of integrin β8 protein expression (Figure 1C), confirming the results seen at the gene level. Conversely, to investigate whether EphB4 over-expression would result in a parallel increase in *ITGB8* expression, and to ensure that these data were not due to siRNA off-target effects, the expression of *ITGB8* in stable 22Rv1-EphB4 over-expressing cells was also determined using qRT-PCR and compared with 22Rv1 cells containing the empty vector (22Rv1-VO). EphB4 over-expression resulted in a significant 2.5 fold increase in *ITGB8* gene levels, but no significant effect was seen on *ITGA3* or *ITGA10* expression (Figure 1D). Again, the increase in gene expression of *ITGB8* correlated with an increase in protein level (Figure 1E). Together, these data suggest that *EPHB4* and *ITGB8* are co-regulated in prostate cancer cells. As integrin β8 has only one known heterodimer partner, integrin αV (ITGAV) [10], we also sought to determine whether over-expression of EphB4 increases the expression of this integrin subunit. *EPHB4* overexpression in 22Rv1 cells significantly increased *ITGAV* gene expression by 1.7 fold (Figure 1F) suggesting an overall increase in the integrin αvβ8 heterodimer complex.

### Knockdown of *ITGB8* suppresses migration and invasion in prostate cancer cells

**Figure 2:**
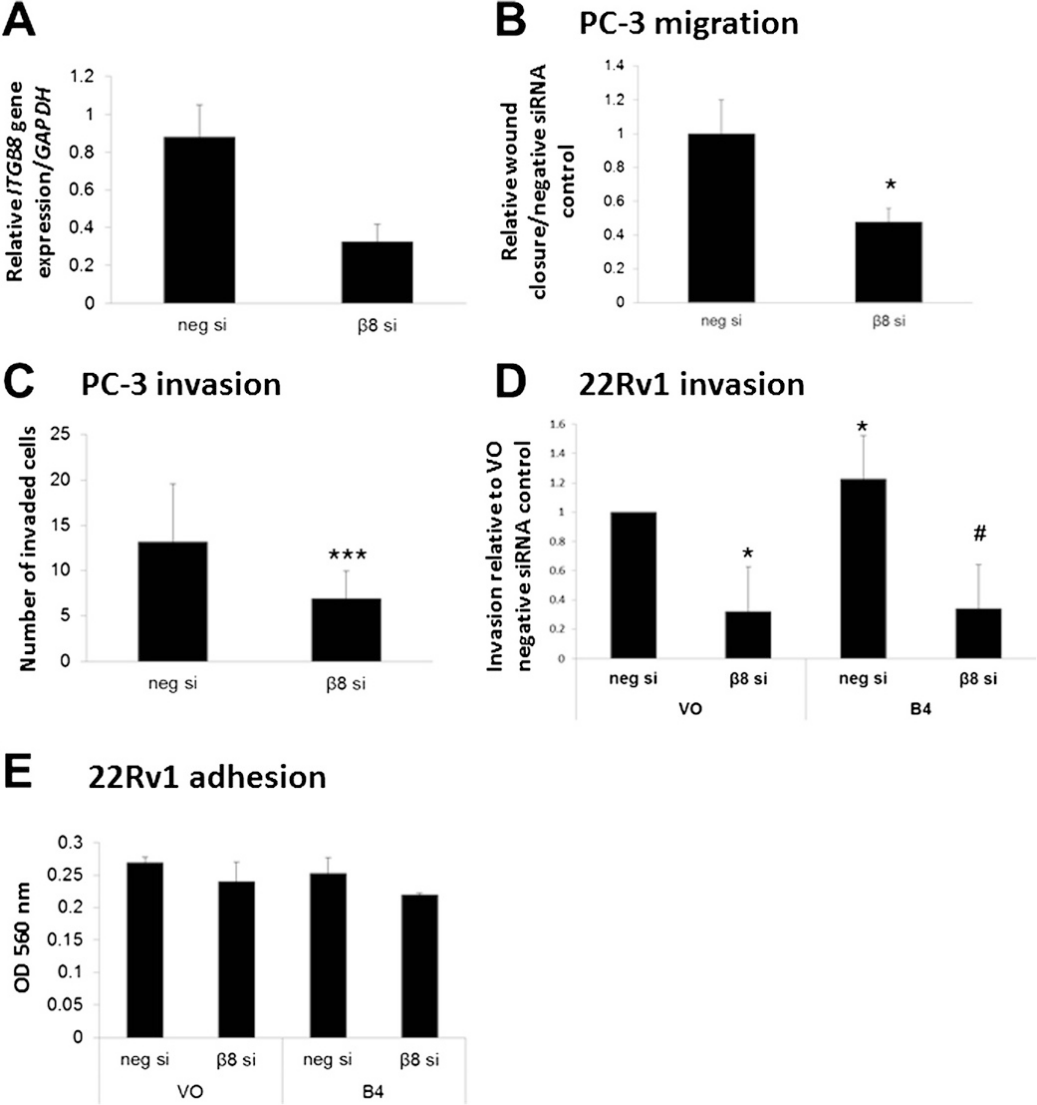
**Knockdown of*ITGB8*results in reduced metastatic potential in prostate cancer cells. A)** Quantitative real-time PCR was carried out to determine knockdown levels of siRNA against *ITGB8*. 100 nM negative non-silencing or *ITGB8* targeting siRNA were transiently transfected into PC-3 cells. RNA was isolated 48 h after transfection, transcribed into cDNA and analyzed for gene expression. *ITGB8* expression is reduced by approximately 60-70%. **B)** PC-3 cells were transiently transfected with 100 nM negative non-silencing (neg si) or *ITGB8* targeting siRNA (β8 si) and 24 h later a scratch wound was applied using the IncuCyte (Essen Bioscience) system and migration was monitored for a further 24 h. Cell migration was reduced following knockdown of *ITGB8*. **C)** PC-3 cells were transiently transfected with 100 nM negative non-silencing (neg si) or *ITGB8* targeting siRNA (β8 si) and subjected to a Matrigel transwell invasion assay. After 22 h of incubation, invaded cells were stained and counted. Cell invasion was reduced following knockdown of *ITGB8*. **D)** 22Rv1-VO (vector only) or 22Rv1–B4 (EphB4 over-expressing) cells were transiently transfected with 100 nM siRNA against *ITGB8* (β8 si) or negative non-silencing siRNA (neg si). An invasion assay was carried out using the Matrigel invasion system and cells were allowed to invade for 22 h. Cells containing the *ITGB8* siRNA showed significantly reduced ability to invade. **E)** 22Rv1-VO (vector only) or 22Rv1–B4 (EphB4 over-expressing) cells were transiently transfected with siRNA against *ITGB8* (β8 si) or negative non-silencing siRNA (neg si) and subjected to an adhesion assay to vitronectin. No significant changes were seen. n = 3 * *p* < 0.01 vs VO negative; # *p* < 0.001 vs B4 negative.

Integrin β8 has been implicated in tumor cell invasiveness [11]. To analyse functional effects of integrin β8 in prostate cancer cells, we employed siRNA knockdown. In PC-3 cells, *ITGB8* knockdown resulted in a 70% decrease in *ITGB8* mRNA (Figure 2A). PC-3 cells transfected with *ITGB8*-targeted siRNA showed a significant decrease in *in vitro* wound healing migration and in Matrigel invasion, when compared with PC-3 cells transfected with negative siRNA (Figure 2B & C). Furthermore, transfection of 22Rv1-VO and 22Rv1-B4 cells with the *ITGB8* siRNA resulted in a significant decrease in Matrigel invasion (Figure 2D). There was no significant difference in the adhesion to vitronectin of 22Rv1-VO or 22Rv1–B4 cells transfected with *ITGB8* siRNA (Figure 2E). Together, these results highlight that integrin β8 plays an important role in prostate cancer cell migration and invasion in both endogenous and exogenously over-expressing EphB4 cell models.

### Correlation between *ITGB8* and *EPHB4* expression levels

**Figure 3:**
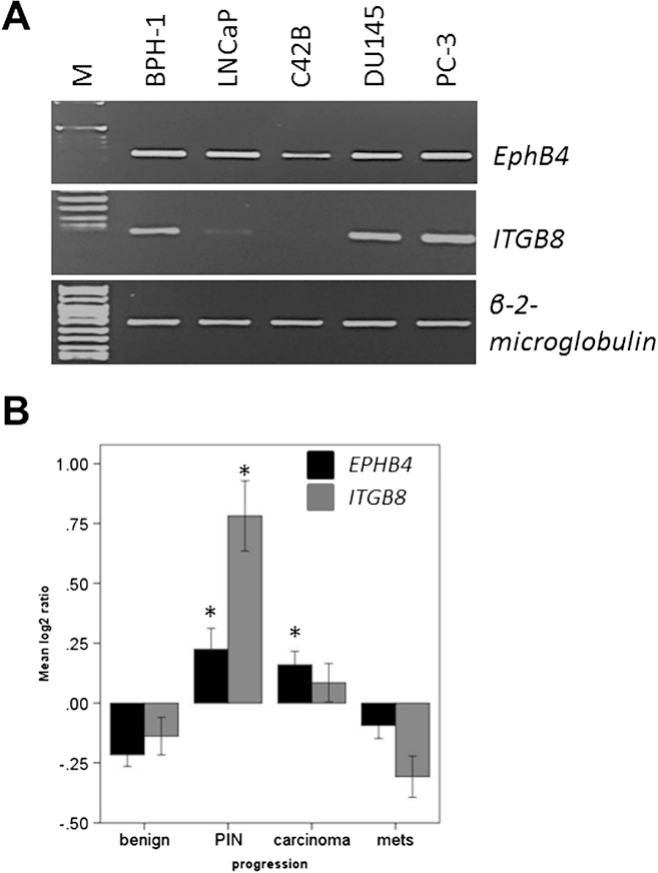
**Integrin expression levels across disease progression. A)** cDNA from several different prostate-derived cell lines was analyzed using semi-quantitative RT-PCR. *GAPDH* amplification was used as a loading control. **B)** Gene expression omnibus dataset GDS3289 investigating prostate cancer progression in LCM-captured clinical samples was interrogated for *EPHB4* and *ITGB8* expression using the Oncomine clinical database (www.oncomine.org). Both genes are significantly elevated in prostatic intraepithelial neoplasia (PIN) and *EPHB4* is significantly upregulated in carcinoma samples compared to benign. * p < 0.001, mets = metastatic disease.

To investigate whether there is a correlation between *ITGB8* and *EPHB4* expression in different prostate cancer cells representing different stages of the disease, several cell lines were subjected to RT-PCR (Figure 3A). All prostate cell lines tested expressed *EPHB4* as expected [5,7]. *ITGB8* was most highly expressed in PC-3 and DU145 cells, followed by BPH-1 and LNCaP cells. The metastatic subclonal line C4-2B derived from LNCaP cells [12] showed no *ITGB8* mRNA expression. To further assess whether *ITGB8* expression could be indicative of clinical disease progression we surveyed the Oncomine database. Analysis of the prostate cancer progression study conducted by Tomlins *et al*. [13] revealed that in benign samples both *EPHB4* and *ITGB8* are expressed at a low level and this increases dramatically and significantly in prostatic intraepithelial neoplasia (PIN) (*ITGB8:* 9.5 fold, p = 1.24 x 10^-4^ and *EPHB4* 2.9 fold, p = 0.001), the precursor for prostate carcinoma [14]. In carcinoma samples the expression of both genes is elevated, with *EPHB4* being significantly up-regulated in comparison to benign tissue samples, but expression of both *ITGB8* and *EPHB4* is much lower than in PIN tissues. Metastatic samples show expression levels similar to benign tissue for both *EPHB4* and *ITGB8*. These results indicate that *EPHB4* and *ITGB8* are concurrently up-regulated in PIN and their expression is progressively lowered with disease advancement. This suggests that both genes may play a role in the onset of prostate cancer.

## Discussion

We have recently shown that EphB4 over-expression leads to a more aggressive phenotype in prostate cancer cells [2]. In this study we set out to investigate the gene expression changes that occurred when *EPHB4* was knocked down in LNCaP prostate cancer cells that are endogenously over-expressing EphB4. Microarray analysis revealed a set of three integrin subunits (α3, α10 and β8) that were de-regulated upon siRNA knockdown of *EPHB4* and qRT-PCR validated two of these (α10 and β8). Over-expression of *EPHB4* led to concomitant and parallel changes in expression and protein levels of ITGB8 but not the other two integrins. In keratinocytes, it has been shown that EphB2-induced reverse signaling down-regulated integrin expression, demonstrating that in other cell contexts other Eph receptors also have the ability to influence integrin expression [15]. Integrins are a family of transmembrane receptors which are primarily involved in cell-extracellular matrix (ECM) adhesion as well as cell-cell interactions. By connecting the actin cytoskeleton to the ECM, integrins are able to regulate attachment, cytoskeletal organization, mechano-sensing, migration, proliferation, differentiation and cell survival [16]. Through their several roles, integrins have been found to be involved in a range of pathological processes including tumor angiogenesis and metastasis [17,18].

There is evidence that Eph/ephrin signaling can influence integrin clustering and in some cases, inhibit integrin downstream signaling [19,20]. Furthermore, ephrin-A1 and EphA2 have both been shown to co-localize with integrin α3 [20-22]. In breast cancer cell lines, EphB4 exogenous overexpression reduces integrin β1 expression resulting in increased migration in a ligand-independent manner [23]. In the current study, validation experiments confirmed that *ITGB8* was significantly down-regulated when *EPHB4* was knocked down and moreover, was also up-regulated when *EPHB4* was over-expressed in prostate cancer cells. This suggests that *EPHB4* and *ITGB8* are potentially transcriptionally co-regulated. In terms of transcriptional regulation, the *ITGB8* promotor contains SP and CRE binding motifs and is regulated by SP1, SP3 and an AP-1 complex [24]. *EPHB4* on the other hand has been shown to be transcriptionally regulated by HoxA9 in endothelial cells [25], but no information is available about the transcription factors involved in regulating expression of *EPHB4* in cancer cells. It would be interesting to investigate whether the transcription factors of the SP family can also influence *EPHB4* expression in prostate cancer and thus be responsible for co-regulating *ITGB8* and *EPHB4*.

Although only limited information is available about the role of integrin β8 in cancer it has been identified as up-regulated in several cancers including head and neck cancer, hepatocellular carcinoma, some ovarian cancer and melanoma cell lines as well as primary non-small lung cancer samples and brain metastases from several epithelial cancers [26-28]. Furthermore, *ITGB8* has been identified as a member of a six-gene expression signature biomarker predicting lung metastasis from breast cancer [29]. In glioblastoma specimens, there appears to be a correlation between low integrin β8 expression and highly angiogenic, but poorly invasive tumors; as well as high integrin β8 expression in low angiogenic and highly invasive tumors (17). This implies that integrin β8 is involved in differential control of angiogenesis *versus* tumor cell invasion in glioblastomas. On the other hand, integrin β8 has been shown to be growth inhibitory in lung cancer cells, yet it is also playing a fundamental role in lung cancer metastasis indicating that this integrin functions in a complex manner in cancer presumably depending on tissue and progression context [30-32]. As the expression of integrin β8 has been found to be increased in metastases from various tumors, including breast and lung, we speculated that integrin β8 could also play a role in prostate cancer cell migration and invasion and we show that silencing of *ITGB8* reduces cell motility [28,29]. In highly invasive glioblastoma tumors, high levels of integrin β8 cooperate with Rho proteins to drive invasion [11]. Eph receptors are also able to activate the Rho pathway to regulate cancer cell migration [33] and thus it is possible that EphB4 and integrin β8 work cooperatively to control cell motility.

By interrogating the Oncomine database we have identified *ITGB8* as being up-regulated in PIN, the precursor to prostate cancer which is puzzling considering its role in metastases. It is possible that integrin β8 is able to impact on different stages of tumor development in different cell populations. In established prostate cancer cell lines we demonstrate that integrin β8 plays a vital role in migration and invasion. These results are in agreement with previous reports describing similar findings in lung cancer and glioblastoma [11,31].

Integrin β8 has been shown to only heterodimerise with the integrin αv subunit and this heterodimer binds to vitronectin [10]. We show here that the αv subunit expression also increases in stably EphB4 over-expressing prostate cancer cells, but no effect on adhesion to vitronectin was seen. Interestingly, transforming growth factor β (TGF-β) has been identified as the only physiologically relevant ligand for integrin αvβ8 and activation of matrix-bound latent TGF-β by integrin αvβ8 results in activation of TGF-β signaling and remodeling of human airway fibroblasts [34]. TGF-β is a well-known cytokine with a variety of physiological functions such as proliferation, differentiation and immune response and in addition it plays a major role in cancer progression by inducing epithelial-to-mesenchymal transition (EMT) in prostate cancer and promoting metastasis to the bone, the final step in prostate cancer progression [35,36]. In our array data *TGFB1* was also found to be significantly upregulated by 2-fold (data not shown). This could indicate that *EPHB4*, *ITGB8* and *TGFB1* are intrinsically regulated in prostate cancer cells, therefore contributing to cancer progression and metastasis through the process of EMT. However, expression analysis of the EMT markers E-cadherin and Snail did not show any significant changes in PC-3 cells transfected with *ITGB8* siRNA (data not shown). Changes in E-cadherin and Snail expression have been reported in lung cancer cells where *ITGB8* was silenced [31].

Cilengitide, a selective antagonist of αvβ3 and vβ5 integrins, is currently in clinical trials for a variety of solid tumors, but so far the results are modest [37-39]. In prostate cancer, no clinical effect was seen [40]. Targeting the αvβ8 integrin in combination with EphB4 targeted therapies may represent a future avenue for prostate cancer therapy.

## Conclusions

Alteration of EphB4 levels (through knockdown or over-expression) concurrently, and in a similar manner, alters the levels of integrin β8. The high level of expression of both *EPHB4* and *ITGB8* in clinical PIN samples suggests that their increased expression is an early event in the development of prostate cancer. This also identifies a new mechanism for EphB4 function in prostate cancer through the regulation of ITGB8, which our results show can contribute to prostate cancer cell motility. Targeting both EphB4 and integrin β8 may provide new options for treating prostate cancer.

## Additional file

Additional file 1: Figure S1. Microarray analysis of de-regulated genes after siRNA knockdown of *EPHB4* in LNCaP prostate cancer cells. Volcano plot representing up-and down-regulated genes (log2 scale, fold change) comparing non-silencing siRNA samples (n = 3) to *EPHB4* knockdown samples (n = 4). The analysis was carried out using Genespring GX11 (Agilent Technologies).
