# Supplementary figures and images for: The tumour-promoting receptor tyrosine kinase, EphB4, regulates expression of Integrin-β8 in prostate cancer cells
Source: BMC Cancer. 2015 Mar 22;15:164. doi: 10.1186/s12885-015-1164-6 (PMC4389669; doi:10.1186/s12885-015-1164-6)

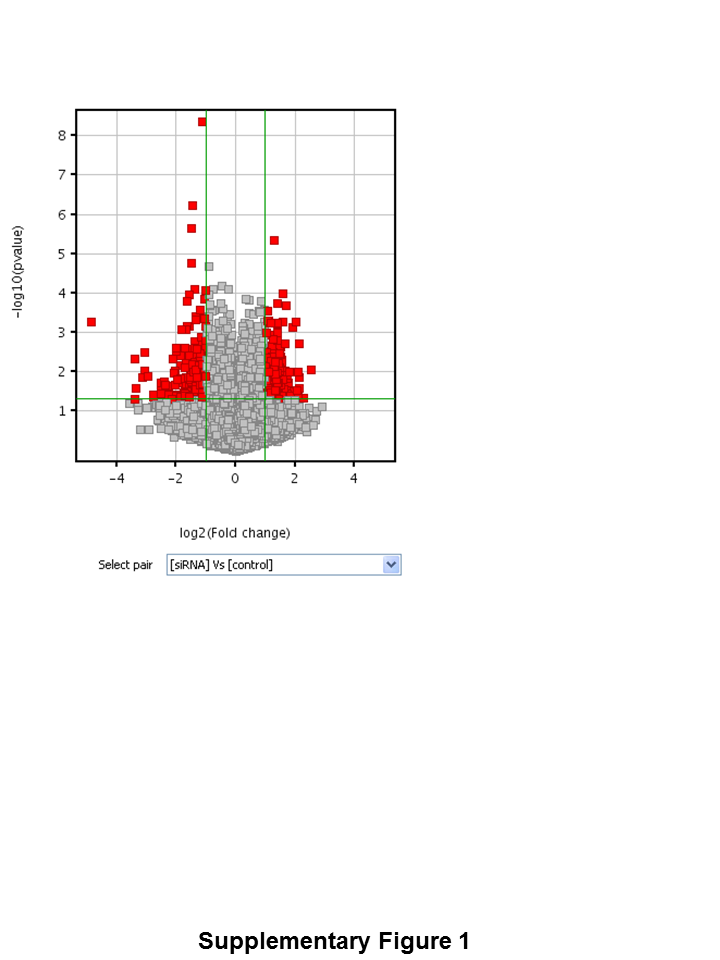

Supplement: Additional file 1: Figure S1. — Microarray analysis of de-regulated genes after siRNA knockdown of EPHB4 in LNCaP prostate cancer cells. Volcano plot representing up-and down-regulated genes (log2 scale, fold change) comparing non-silencing siRNA samples (n = 3) to EPHB4 knockdown samples (n = 4). The analysis was carried out using Genespring GX11 (Agilent Technologies). [file 12885_2015_1164_MOESM1_ESM.tiff]
